# Supplementary material for: Equity in Vaccine Trials for Higher Weight People? A Rapid Review of Weight-Related Inclusion and Exclusion Criteria for COVID-19 Clinical Trials
Source: Vaccines (Basel). 2021 Dec 11;9(12):1466. doi: 10.3390/vaccines9121466 (PMC8708246; doi:10.3390/vaccines9121466)
Supplement: Supplementary file 1 [file vaccines-09-01466-s001.zip › vaccines-1486337-supplementary.pdf]

vaccines

MDPI

Supplementary Material: Table S1: Number of registered trial locations, by country.; Table S2: Vaccine Platforms.; Table S3: Included Trial Protocols.

Table S1. Number of registered trial locations, by country.

Country	Number of Trials
China	44
United States of America	36
Brazil	13
Japan	13
Russia	12
Belgium	11
United Kingdom	11
Australia	10
Germany	9
Republic of Korea	9
Canada	8
India	7
Not specified/pending	7
South Africa	8
Peru	7
Cuba	7
Iran	6
Turkey	6
Argentina	5
Colombia	5
France	5
Taiwan	5
Chile	4
Indonesia	4
Italy	4
Mexico	4
Netherlands	4

Panama	3
Singapore	3
Spain	3
United Arab Emirates	3
Vietnam	2
Egypt	2
Hong kong	2
Israel	2
Kazakhstan	2
Pakistan	2
Philippines	2
Thailand	2
Austria	1
Azerbaijan	1
Bahrain	1
Belarus	1
Denmark	1
Dominican Republic	1
Guantanamo	1
Ecuador	1
Honduras	1
Jordan	1
Sweden	1
Kenya	1
Malaysia	1
Morocco	1
Nepal	1
New Zealand	1
Poland	1
Switzerland	1
Uzbekistan	1
Havana	1
Venezuela	1

Table S2. Vaccine Platforms.

Vaccine Platform	Number of Trials
DNA	19 (7.63%)
RNA	53 (21.29%)
Inactivated Virus	43 (17.27%)
Live attenuated Virus	1 (0.40%)
Protein Subunit	60 (24.10%)
Virus-like Particle Not Otherwise Specified	6 (2.41%)
Replicating Vector Not Otherwise Specified	11 (4.42%)
Non-Replicating Vector Not Otherwise Specified	53 (21.29%)
Other/Unknown	3 (1.20%)

Table S3. Included Trial Protocols.

Record Title	First Author/Principal Investigator/Contact Person	Start Date	Trial Phase	Status of Trial on Date of Data Analysis	Place of Trial	Age Range	Participant Health Status	Includes BMI >30
https://pactr.samrc.ac.za/TrialDisplay.aspx?TrialID=13475	Videlis Nduba	01/12/2020	Not specified	Withdrawn	Kenya	≥18	Healthy	Not specified
https://clinicaltrials.gov/ct2/show/NCT04794946	Dr Shantan Venishetty, MD	19/03/2021	Not specified	Recruiting	India	18-60	Cirrhosis	Not specified
https://clinicaltrials.gov/ct2/show/NCT04862806	Tamar Tadmor	01/02/2021	Not specified	Recruiting	Israel	≥18	Chronic Lymphocytic Leukaemia	Not specified
https://clinicaltrials.gov/ct2/show/NCT04715438	E G de Vries, MD, PhD	08/01/2021	Not specified	Recruiting	Netherlands	≥18	Cancer	Not specified
https://clinicaltrials.gov/ct2/show/NCT04844489	Anne-Geneviève MARCELIN, MD, PhD	16/04/2021	Not specified	Recruiting	France	≥18	Immunocompromised	Not specified
http://www.chictr.org.cn/historyversionpuben.aspx?regno=ChiCTR2100045109	Yihao Liu	09/04/2021	Not specified	Pending	China	18-59	Healthy	Not specified
http://www.chictr.org.cn/historyversionpuben.aspx?regno=ChiCTR2100041704	Jing Chen	28/09/2020	Not specified	Completed	China	18-59	Healthcare workers	Not specified

http://www.chictr.org.cn/historyversionpuben.aspx?regno=ChiCTR2100041705	Jing Chen	28/09/2020	Not specified	Recruiting	China	18-59	Airline Staff	Not specified
http://www.chictr.org.cn/historyversionpuben.aspx?regno=ChiCTR2100041706	Jing Chen	28/09/2020	Not specified	Recruiting	China	18-59	Security Personnel	Not specified
ChiCTR2000034112	Lin Yuan	25/06/2020	Phase I	Pending	China	18-80	Healthy	No (>18/<30)
https://ClinicalTrials.gov/show/NCT04522089	Ellie Chen	24/08/2020	Phase I	Recruiting	Taiwan	20-60	Healthy	Yes (>18/<35)
https://ClinicalTrials.gov/show/NCT04690387	Robert O Dillman, MD	7/12/2020	Phase I	Completed	Indonesia	≥18	Relatively Healthy	Yes
https://ClinicalTrials.gov/show/NCT04679909	Altimmune, Inc	01/02/2021	Phase I	Recruiting	USA	18-55	Healthy	No (<30)
https://ClinicalTrials.gov/show/NCT04550351	Anhui Zhifei, Longcom Biologic Pharmacy Co., Ltd.	19/08/2020	Phase I	Active, not recruiting	China	≥60	Healthy	No
https://ClinicalTrials.gov/show/NCT04445194	Anhui Zhifei,Longcom Biologic Pharmacy Co., Ltd.	22/06/2020	Phase I	Recruiting	China	18-59	Healthy	No (18-28)
ChiCTR2000035691	Anhui Zhifei, Longcom Biologic Pharmacy Co., Ltd.	20/08/2020	Phase I	Recruiting	China	≥60	Healthy	No
ChiCTR2000038804	Pan Hongxing	07/10/2020	Phase I	Not yet recruiting	China	≥18	Healthy	Not specified
https://ClinicalTrials.gov/show/NCT04523571	Jingxin Li	21/08/2020	Phase I	Active, not recruiting	China	18-85	Healthy	No (18-30)
ChiCTR2000030906	Zhu Fengcai	17/03/2020	Phase I	Recruiting	China	18-60	Healthy	No
https://ClinicalTrials.gov/show/NCT04313127	Zhu Fengcai	18/03/2020	Phase I	Active, not recruiting	China	18-60	Healthy	No
https://ClinicalTrials.gov/show/NCT04566276	Sivaporn Gatechompol, MD	28/09/2020	Phase I	Not yet recruiting	Thailand	18-75	Healthy	No
https://ClinicalTrials.gov/show/NCT04405908	Peter Richmond	28/05/2020	Phase I	Completed	Australia	18-75	Healthy	Yes (18.5-35)
https://ClinicalTrials.gov/show/NCT04619628	Daryl Bendel, MD	06/11/2020	Phase I	Active, not recruiting	United Kingdom	18-30	Healthy	No. >50kg. BMI 18-28. Up to BMI 30 if muscular physique at investigator's discression.

https://ClinicalTrials.gov/show/NCT04449276	CureVac AG	26/06/2020	Phase I	Active, not recruiting	Belgium, Germany	18-60	Healthy	No
https://ClinicalTrials.gov/show/NCT04591184	Sally Jones, Gregory Reid	19/10/2020	Phase I	Recruiting	Canada	18-85	Healthy	No (<30)
https://ClinicalTrials.gov/show/NCT04691947	Zafer Sezer	31/12/2020	Phase I	Recruiting	Turkey	18-55	Healthy	No (18.5-30)
https://ClinicalTrials.gov/show/NCT04591717	Julian Blake, Deborah Fridman	19/10/2020	Phase I	Recruiting	US	18-55	Healthy	No
https://ClinicalTrials.gov/show/NCT04710303	Amy Ward	14/01/2021	Phase I	Recruiting	South Africa	18-50	Healthy	No
ISRCTN17072692	Mr James Fletcher	22/05/2020	Phase I	Ongoing, no longer recruiting	United Kingdom	18-75	Healthy	Not specified
https://ClinicalTrials.gov/show/NCT04685603	Muhammad Karyana, Dr.	28/12/2020	Phase I	Recruiting	Indonesia	≥18	Healthy	Not specified
ChiCTR2000038152	Jing Zhang	11/09/2020	Phase I	Active, not recruiting	China	18-59	Healthy	No (18-30)
https://ClinicalTrials.gov/show/NCT04336410	Mammen P. Mammen	07/04/2020	Phase I	Active, not recruiting	United States	≥18	Healthy	No
https://ClinicalTrials.gov/show/NCT04497298	Odile LAUNAY, MD, PhD	04/08/2020	Phase I	Active, not recruiting	France, Belgium	18-55	Healthy	No (<30)
https://ClinicalTrials.gov/show/NCT04552366	S Wu	17/09/2020	Phase I	Active, not recruiting	China	≥18	Healthy	Not specified
IFV/COR/05	Sonia Perez Rodriguez	19/10/2020	Phase I	Completed	Cuba	19-59	Healthy	No (18.5-29.9)
IFV/COR/06	Maria Eugenia Toledo Romani	29/10/2020	Phase I	Completed	Cuba	19-59	Healthy	No
https://ClinicalTrials.gov/show/NCT04509947	Janssen Pharmaceutical K.K.	12/08/2020	Phase I	Active, not recruiting	Japan	≥20	Healthy	Yes (<40)
https://ClinicalTrials.gov/show/NCT04436276	Kathryn E. Stephenson	18/06/2020	Phase I	Completed	Belgium, US	≥18	Healthy	No (<30)
https://ClinicalTrials.gov/show/NCT04568811	Jiangsu Province Centers for Disease Control and Prevention	29/09/2020	Phase I	Active, not recruiting	China	18-60	Healthy	Not specified
https://ClinicalTrials.gov/show/NCT04636333	Fanyue Meng, Doctor	19/11/2020	Phase I	Recruiting	China	≥18	Healthy	Yes (<35)

https://ClinicalTrials.gov/show/NCT04530656	Fengcai Zhu, Doctor	28/08/2020	Phase I	Active, not recruiting	China	18-55	Healthy	No (<30)
https://ClinicalTrials.gov/show/NCT04450004	Brian J Ward	10/07/2020	Phase I	Active not recruiting	Canada	18-55	Healthy	No (18-30)
https://ClinicalTrials.gov/show/NCT04487210	SM Hsieh	7/10/2020	Phase I	Active not recruiting	Taiwan	20-50	Healthy	No
https://ClinicalTrials.gov/show/NCT04569786	N Kreuzberger	29/10/2020	Phase I	Terminated	United States	≥18	Relatively Healthy	No (<30)
https://ClinicalTrials.gov/show/NCT04283461	Lisa A. Jackson	16/03/2020	Phase I	Active not recruiting	United States	18-99	Relatively Healthy	Yes (18-35)
ChiCTR2000034825	Zhu Fengcai	20/07/2020	Phase I	Recruiting	China	18-55	Healthy	No (18-30)
https://ClinicalTrials.gov/show/NCT04627675	Tara Foote	30/12/2020	Phase I	Active not recruiting	United States	≥18	Healthy	Yes (<35)
https://ClinicalTrials.gov/show/NCT04528641	Simone Lanini	10/08/2020	Phase I	Active not recruiting	Italy	18-55, 65-85	Healthy	No
https://ClinicalTrials.gov/show/NCT04334980	Paul Griffin	2/11/2020	Phase I	Active not recruiting	Australia	≥18	Healthy	Yes (18-32)
https://ClinicalTrials.gov/show/NCT04495933	Paul Griffin	13/07/2020	Phase I	Recruiting	Australia	≥18	Healthy	Yes (18-34)
https://ClinicalTrials.gov/show/NCT04545749	Chang-Yi Wang	25/09/2020	Phase I	Active not recruiting	Taiwan	20-55	Healthy	No
https://ClinicalTrials.gov/show/NCT04569383	Marylyn M Addo	5/10/2020	Phase I	Recruiting	Germany	18-55	Healthy	No
https://ClinicalTrials.gov/show/NCT04546841	Helmut Salih	27/11/2020	Phase I	Recruiting	Germany	≥18	Healthy	No
ChiCTR2000037782	Fengcai Zhu	01/09/2020	Phase I	Not yet recruiting	China	≥18	Healthy	Not specified
https://ClinicalTrials.gov/show/NCT04702178	Joanne M Langley	10/02/2021	Phase I	Recruiting	Canada	≥18	Healthy	Yes
https://ClinicalTrials.gov/show/NCT04563702	Shaily J Garg	21/09/2020	Phase I	Active not recruiting	United States	18-54	Healthy	No
ChiCTR2000037518	Fengcai Zhu	28/08/2020	Phase I	Pending	China	≥18	Relatively Healthy	No (18.5-30)
http://www.chictr.org.cn/historyversionpuben.aspx?regno=ChiCTR2100045984	Li Lanjuan, Chen Guiling	25/03/2021	Phase I	Recruiting	China	≥18	Healthy	Not specified

https://clinicaltrials.gov/ct2/show/NCT04785144	National Institute of Allergy and Infectious Diseases (NIAID)	29/03/2021	Phase I	Recruiting	USA	≥18	Healthy	Yes (<40)
http://www.chictr.org.cn/historyversionpuben.aspx?regno=ChiCTR2100045108	Xu Zhonghui	22/02/2021	Phase I	Recruiting	China	≥18	Healthy	No (18-28)
https://clinicaltrials.gov/ct2/show/NCT04758273	Beijing Minhai Biotechnology Co., Ltd	7/10/2020	Phase I	Active, not recruiting	China	≥18	Healthy	Not specified
https://clinicaltrials.gov/ct2/show/NCT04751682	AO Hassan	01/03/2021	Phase I	Active, not recruiting	India	18-60	Healthy	Yes (18-35)
https://clinicaltrials.gov/ct2/show/NCT04818801	Christopher Wynne	01/04/2021	Phase I	Not yet recruiting	New Zealand	18-80	Healthy	Yes (18-35)
https://clinicaltrials.gov/ct2/show/NCT04742842	Nicholas WOOD, MB BS FRACP PhD.	15/02/2021	Phase I	Not yet recruiting	Australia	18-75	Healthy	Yes (18-35)
https://clinicaltrials.gov/ct2/show/NCT04776317	Daniel F. Hoft	25/03/2021	Phase I	Recruiting	USA	≥18	Healthy	No
https://en.irct.ir/trial/54133	Ahmad Karimi Rahjerdi	10/03/2021	Phase I	Recruitment complete	Iran	18-55	Healthy	Not specified
https://www.irct.ir/trial/52975	Ali Eshagi	29/01/2021	Phase I	Recruitment complete	Iran	18-55	Healthy	Yes (17-35)
https://clinicaltrials.gov/ct2/show/NCT04798001	Oliver Medzihradsky, MD MPH MS	12/04/2021	Phase I	Recruiting	USA	18-69	Healthy	Not specified
https://clinicaltrials.gov/ct2/show/NCT04639466	John Zaia	11/12/2020	Phase I	Recruiting	USA	18-55	Healthy	Yes (<35)
https://clinicaltrials.gov/ct2/show/NCT04813796	Dr Krishna Mohan	11/03/2021	Phase I	Recruiting	USA	18-55	Healthy	Yes (18-35)
https://clinicaltrials.gov/ct2/show/NCT04809389	Ivan Fan-ngai Hung	29/03/2021	Phase I	Recruiting	Hong Kong	18-55	Healthy	No (18.5-25)
https://clinicaltrials.gov/ct2/show/NCT04871737	Samuel Ponce de Leon, MD	01/05/2021	Phase I	Not yet recruiting	Mexico	18-55	Healthy	No
https://clinicaltrials.gov/ct2/show/NCT04844268	Dr Bruna Machado, Carolina Macedo, PhD	01/05/2021	Phase I	Not yet recruiting	Pending	18-55	Healthy	Yes (<32)
https://clinicaltrials.gov/ct2/show/NCT04784767	Paul T Scott, M.D	05/04/2021	Phase I	Recruiting	USA	18-55	Healthy	Yes (18-35)

https://clinicaltrials.gov/ct2/show/NCT04732468	Lennie Sender, MD	24/02/2021	Phase I	Recruiting	USA	18-55	Healthy	No
https://clinicaltrials.gov/ct2/show/NCT04765436	Dr. Piyush Patel, MD, FRCP	14/01/2021	Phase I	Recruiting	Canada	18-64	Healthy	No
https://en.irct.ir/trial/52701	Mohammadreza Hosseinpour	21/12/2020	Phase I	Recruitment complete	Iran	18-50	Healthy	Yes (<40)
https://clinicaltrials.gov/ct2/show/NCT04816019	Alexander Douglas, Dr	01/04/2021	Phase I	Enrolling by invitation	UK	18-40	Healthy	Yes (<50)
https://clinicaltrials.gov/ct2/show/NCT04760743	Myoung-Don Oh	17/12/2020	Phase I	Active, not recruiting	Republic of Korea	19-55	Healthy	No
https://clinicaltrials.gov/ct2/show/NCT04866069	Osman ERGANIS, PhD	25/04/2021	Phase I	Recruiting	Turkey	18-45	Healthy	Yes (18-35)
https://clinicaltrials.gov/ct2/show/NCT04885361	Ose immunotherapeutics	10/05/2021	Phase I	Not yet recruiting	Belgium	18-45	Healthy	No
https://clinicaltrials.gov/ct2/show/NCT04839146	Merel J. Smit, MD	11/03/2021	Phase I	Recruiting	Netherlands	18-55	Healthy	Yes (<35)
https://clinicaltrials.gov/ct2/show/NCT04758962	GlaxoSmithKline	15/02/2021	Phase I	Recruiting	USA	18-50	Healthy	No
https://clinicaltrials.gov/ct2/show/NCT04839042	Jonathan Stocker, Ph.D.	01/06/2021	Phase I	Not yet recruiting	Australia	18-55	Healthy	Yes (18-32)
https://clinicaltrials.gov/ct2/show/NCT04453852	David Gordon	30/06/2020	Phase I	Completed	Australia	18-65	Healthy	Not specified
https://clinicaltrials.gov/ct2/show/NCT04838080	Turgay Celik, Prof	19/03/2021	Phase I	Recruiting	Turkey	18-55	Healthy	No
https://clinicaltrials.gov/ct2/show/NCT04818281	Recep S SENTURK	27/03/2021	Phase I	Recruiting	Turkey	18-59	Healthy	Yes (18-35)
https://en.irct.ir/trial/54885	Mohammadreza Hosseinpour	13/03/2021	Phase I	Recruitment complete	Iran	51-75	Healthy	Yes (<40)
https://ClinicalTrials.gov/show/NCT04386252	Candace Hsieh, PhD	01/06/2021	Phase I/II	Not yet recruiting	Australia	≥18	Relatively Healthy	Yes
jRCT2051200085	Hiromi Rakugi	19/11/2020	Phase I/II	Not recruiting	Japan	20-75	Healthy	Not specified
https://ClinicalTrials.gov/show/NCT04527081	AnGes, Inc.	31/08/2020	Phase I/II	Active, not recruiting	Japan	20-65	Healthy	Not specified
https://ClinicalTrials.gov/show/NCT04463472	AnGes, Inc.	29/06/2020	Phase I/II	Active, not recruiting	Japan	20-65	Healthy	Not specified

https://ClinicalTrials.gov/show/NCT04480957	Jenny Low Guek Hong, MD	08/04/2020	Phase I/II	Recruiting	Singapore	21-55, 56-80	Healthy	Yes (18-35)
https://ClinicalTrials.gov/show/NCT04568031	M Asano	23/08/2020	Phase I/II	Active, not recruiting	Japan	≥18	Healthy	Not specified
https://ClinicalTrials.gov/show/NCT04684446	Mikhail Samsonov	30/03/2021	Phase I/II	Not yet recruiting	Russian Federation	18-130	Healthy	Not specified
https://ClinicalTrials.gov/show/NCT04380701	AB Vogel	8/04/2020	Phase I/II	Recruiting	Germany	18-85	Healthy/Immunocompromised	Yes (18-35)
https://ClinicalTrials.gov/show/NCT04537949	BioNTech SE	3/09/2020	Phase I/II	Active, not recruiting	Germany	18-85	Healthy	Yes (18-35)
https://ClinicalTrials.gov/show/NCT04588480	BioNTechSE	19/10/2020	Phase I/II	Active, not recruiting	Japan	20-85	Healthy	Not specified
CTRI/2020/11/029032	Dr Subhash Thuluva	10/11/2020	Phase I/II	Active, not recruiting	India	18-65	Healthy	Yes (<40)
https://ClinicalTrials.gov/show/NCT04398147	Scott A Halperin, MD	21/05/2020	Phase I/II	Not yet recruiting	Canada	18-84	Healthy	Yes (18-35)
RPCEC00000345	Carlos Gonzalez Delgado	7/12/2020	Phase I/II	unknown	Cuba	19-80	Healthy	Yes (18.5-35)
RPCEC00000346	Maria Ricardo Cobas	7/12/2020	Phase I/II	unknown	Cuba	19-80	Healthy	Yes (18.5-35)
https://ClinicalTrials.gov/show/NCT04412538	Qihan Li, PhD	2/06/2020	Phase I/II	Recruiting	China	18-59	Healthy	No (18-30)
https://ClinicalTrials.gov/show/NCT04527575	Vladimir I. Kuzubov	26/08/2020	Phase I/II	Active, not recruiting	Russia	18-60	Healthy	No
https://ClinicalTrials.gov/show/NCT04436471	Denis Y Logunov	18/06/2020	Phase I/II	Completed	Russia	18-60	Healthy	No
https://ClinicalTrials.gov/show/NCT04437875	Lola Morozova	18/06/2020	Phase I/II	Completed	Russia	18-60	Healthy	No
https://ClinicalTrials.gov/show/NCT04713488	Gamaleya Research Institute of Epidemiology and Microbiology	19/01/2021	Phase I/II	Active, not recruiting	Russia	18-111	Healthy	Not specified
https://ClinicalTrials.gov/show/NCT04673149	Joel N Maslow	17/12/2020	Phase I/II	Recruiting	Korea	19-65	Healthy	Not specified
https://ClinicalTrials.gov/show/NCT04445389	Yoon-Jeong Choi	24/06/2020	Phase I/II	Recruiting	Korea	18-50	Healthy	No (Weight 50-90kg, BMI 18-28)
https://ClinicalTrials.gov/show/NCT04715997	JungWon Woo	20/01/2021	Phase I/II	Recruiting	Korea	18-55	Healthy	Not specified

IFV/COR/04	Sonia Perez Rodriguez	13/08/2020	Phase I/II	Recruiting	Cuba	19-80	Healthy	No (18-29.9)
https://ClinicalTrials.gov/show/NCT04608305	Hadar Marcus, Dr.	29/10/2020	Phase I/II	Recruiting	Israel	18-85	Healthy	Not specified
https://ClinicalTrials.gov/show/NCT04473690	Oscar Guzman	30/12/2020	Phase I/II	Recruiting	US	18-85	Healthy	No (<30)
https://ClinicalTrials.gov/show/NCT04498247	Merck Sharp & Dohme Corp.	27/08/2020	Phase I/II	Terminated	United States, Austria, Belgium	≥18	Healthy	No (<30)
https://ClinicalTrials.gov/show/NCT04368988	Cheryl Keech	25/05/2020	Phase I/II	Active not recruiting	Australia, United States	18-59 (phase I), 18-84 (phase II)	Healthy	Yes (17-35)
https://ClinicalTrials.gov/show/NCT04530357	Ilyas Kulmagambetov	19/09/2020	Phase I/II	Completed	Kazakhstan	18-100	Healthy	Not specified
https://ClinicalTrials.gov/show/NCT04537208	Sanofi Pasteur	03/09/2020	Phase I/II	Active not recruiting	United States	≥18	Healthy	Not specified
ACTRN12620000817943	Greg Plunkett	22/08/2020	Phase I/II	Recruiting	Australia	18-79	Healthy	Yes (<35)
jRCT2051200092	Nagata Tsutae	11/12/2020	Phase I/II	Recruiting	Japan	20-64	Healthy	No (Phase I 18.5-25, phase II 18.5-30)
https://ClinicalTrials.gov/show/NCT04383574	Yuliang Zhao	22/05/2020	Phase I/II	Completed	China	>60	Healthy	Not specified
https://ClinicalTrials.gov/show/NCT04352608	Fengcai Zhu	16/04/2020	Phase I/II	Completed	China	18-59	Healthy	Not specified
https://ClinicalTrials.gov/show/NCT04712110	Takeda	24/02/2021	Phase I/II	Active not recruiting	Japan	>20	Healthy	No (<30)
https://ClinicalTrials.gov/show/NCT04677660	Takeda	21/01/2021	Phase I/II	Active not recruiting	Japan	>20	Healthy	No (<30)
https://ClinicalTrials.gov/show/NCT04681092	Schelto Kruijff	12/04/2021	Phase I/II	Enrolling by invitation	Netherlands	18-65	Healthy	No
https://ClinicalTrials.gov/show/NCT04324606	Andrew Pollard	23/04/2020	Phase I/II	Active not recruiting	United Kingdom	18-55	Healthy	Yes (18-40)
https://ClinicalTrials.gov/show/NCT04444674	Shabir A Madhi	24/06/2020	Phase I/II	Active not recruiting	South Africa	18-65	Healthy and HIV+ individuals	Yes (<40)
https://ClinicalTrials.gov/show/NCT04671017	Valneva Austria GmbH	16/12/2020	Phase I/II	Active not recruiting	United Kingdom	18-55	Healthy	No (18-30)

https://clinicaltrials.gov/ct2/show/NCT04840992	Fengcai Zhu, MSD	20/04/2021	Phase I/II	Not yet recruiting	China	≥18	Healthy	Not specified
https://clinicaltrials.gov/ct2/show/NCT04773665	Joanne M Langley MD	15/03/2021	Phase I/II	Active, not recruiting	Canada	≥18	Healthy	No (<30)
https://clinicaltrials.gov/ct2/show/NCT04843722	Lennie Sender, MD	01/05/2021	Phase I/II	Recruiting	USA	18-80	Healthy	Yes (<35)
https://clinicaltrials.gov/ct2/show/NCT04845191	Lennie Sender, MD	01/05/2021	Phase I/II	Not yet recruiting	USA	18-80	Healthy	Yes (<35)
https://clinicaltrials.gov/ct2/show/NCT04764422	Punnee Pitisuttithum, M.D	20/03/2021	Phase I/II	Recruiting	Thailand	18-75	Healthy	Not specified
https://clinicaltrials.gov/ct2/show/NCT04830800	Be V Le, Thang C Tran	10/03/2021	Phase I/II	Recruiting	Vietnam	18-75	Healthy	Not specified
https://clinicaltrials.gov/ct2/show/NCT04798027	Sanofi Pasteur	12/03/2021	Phase I/II	Recruiting	USA	≥18	Healthy	Not specified
https://clinicaltrials.gov/ct2/show/NCT04750343	Hee Jin Cheong	03/02/2021	Phase I/II	Recruiting	Republic of Korea	19-85	Healthy	No (18-30)
https://clinicaltrials.gov/ct2/show/NCT04783311	YJ Lee	23/02/2021	Phase I/II	Recruiting	Republic of Korea	19-75	Healthy	Not specified
https://clinicaltrials.gov/ct2/show/NCT04742738	Yoonyeong Lee	20/01/2021	Phase I/II	Recruiting	Republic of Korea	19-85	Healthy	No (18-30)
https://jrct.niph.go.jp/en-latest-detail/jRCT2071200106	Oda Yoshiaki	02/03/2021	Phase I/II	Recruiting	Japan	≥20	Healthy	Not specified
https://clinicaltrials.gov/ct2/show/NCT04447781	Myoung-don Oh, Eu Suk Kim	15/07/2020	Phase I/II	Recruiting	Republic of Korea	19-64	Healthy	No (18-30)
https://www.clinicaltrialsregister.eu/ctr-search/trial/2020-003734-20/IT#E	OPIS s.r.l.	15/02/2021	Phase I/II	Ongoing	Italy	≥18	Healthy	No
https://clinicaltrials.gov/ct2/show/NCT04788459	Luigi Aurisicchio	25/02/2021	Phase I/II	Recruiting	Italy	18-65	Healthy	No
https://clinicaltrials.gov/ct2/show/NCT04821674	Daiichi Sankyo, Inc.	15/03/2021	Phase I/II	Recruiting	Japan	20-74	Healthy	No (17.5-30)

http://ctri.nic.in/Clinicaltrials/pmaindet2.php?trialid=53226&EncHid=&userName=covid%20vaccine	Dr Ravindra Mittal	19/03/2021	Phase I/II	Recruiting	India	18-60	Healthy	Not specified
https://clinicaltrials.gov/ct2/show/NCT04760730	Mikhail Samsonov	17/03/2021	Phase I/II	Not yet recruiting	Pending	≥18	Healthy	Not specified
https://clinicaltrials.gov/ct2/show/NCT04863131	Yohei Doi, MD	28/04/2021	Phase I/II	Recruiting	Japan	20-55	Healthy	Not specified
https://ClinicalTrials.gov/show/NCT04666012	Cheong Heejin, Ph.D	14/12/2020	Phase I/IIa	Recruiting	Korea	19-64	Healthy	No
ChiCTR2000039212E	Lin Yuan	28/10/2020	Phase Ib	Recruiting	China	18-59	Healthy	No (>18/<30)
https://ClinicalTrials.gov/show/NCT04470609	Qihan Li, Professor, Chinese Academy of Medical Sciences	14/07/2020	Phase Ib/IIb	Enrolling by invitation	China	⩾60	Healthy	No (18-30
https://ClinicalTrials.gov/show/NCT04466085	S Yang	12/07/2020	Phase II	Active, not recruiting	China	18-59	Healthy	No (18-28)
https://ClinicalTrials.gov/show/NCT04668339	Arcturus Therapeutics, Inc.	07/01/2021	Phase II	Active, not recruiting	USA, Singapore	≥18	Healthy	No
https://ClinicalTrials.gov/show/NCT04649021	BioNTechSE	2/12/2020	Phase II	Active, not recruiting	China	18-85	Healthy	Not specified
ChiCTR2000031781	Zhu Fengcai	8/04/2020	Phase II	Pending	China	≥18	Healthy	No
https://ClinicalTrials.gov/show/NCT04515147	CureVac AG	17/08/2020	Phase II	Active, not recruiting	Panama, Peru	≥18	Healthy or well controlled chronic health condition	Yes (≥18.0 ≤32.0)
https://ClinicalTrials.gov/show/NCT04587219	Nikita Lomakin	14/10/2020	Phase II	Active, not recruiting	Russia	60-111	Those without "severe comorbid disease"	Not specified
ChiCTR2000040146	Fengcai Zhu	22/11/2020	Phase II	Active, not recruiting	China	18-95	Healthy	Yes (18-35)
https://ClinicalTrials.gov/show/NCT04341389	Fengcai Zhu	10/04/2020	Phase II	Completed	China	≥18	Healthy	No (18.5-30)
IFV/COR/08	Maria Eugenia Toledo Romani	17/12/2020	Phase II	Completed	Cuba	19-80	Healthy	Yes (18.5-34.9)
https://ClinicalTrials.gov/show/NCT04640402	Fanyue Meng, Doctor	17/11/2020	Phase II	Recruiting	China	18-85	Healthy	Not specified

https://ClinicalTrials.gov/show/NCT04718467	Fanyue Meng, Doctor	01/02/2021	Phase II	Not yet recruiting	China	18-85	Healthy	Not specified
https://ClinicalTrials.gov/show/NCT04695652	Szu-Min Hsieh	30/12/2020	Phase II	Active not recruiting	Taiwan, Vietnam	20-65	Relatively Healthy	Not specified
https://ClinicalTrials.gov/show/NCT04405076	L Chu	29/05/2020	Phase II	Active not recruiting	United States	≥18	Healthy	No (18-30)
https://ClinicalTrials.gov/show/NCT04533399	Shabir A Madhi	17/08/2020	Phase II	Recruiting	South Africa	18-84	Relatively Healthy	Yes (17-40)
https://ClinicalTrials.gov/show/NCT04686773	Mikhail Samsonov	10/02/2021	Phase II	Not yet recruiting	Azerbaijan	18-100	Relatively Healthy	Not specified
ChiCTR2000039994	Fengcai Zhu	17/11/2020	Phase II	Recruiting	China	18-85	Relatively Healthy	Not specified
http://www.chictr.org.cn/historyversionpuben.aspx?regno=ChiCTR2100045107	Xu Zhonghui	28/03/2021	Phase II	Pending	China	≥18	Healthy	Not specified
https://www.isrctn.com/ISRCTN69254139	Prof. Matthew Snape	08/02/2021	Phase II	No longer recruiting	UK	≥50	Healthy & mild-mod comorbidity	Not specified
https://clinicaltrials.gov/ct2/show/NCT04762680	Sanofi Pasteur	24/02/2021	Phase II	Active, not recruiting	USA, Honduras	≥18	Healthy	Not specified
https://www.ins.gob.pe/ensayosclinicos/rpec/recuperarECPBNuevoEN.asp?numec=054-20	Elizabeth Rospigliosi Lopez	17/08/2020	Phase II	Enrollment closed	Brazil, Panama, Peru	≥18	Healthy	No (<30)
https://clinicaltrials.gov/ct2/show/NCT04860739	Cristobal Belda Iniesta MD	24/04/2021	Phase II	Active, not recruiting	Spain	18-60	Healthy or stable clinical condition	Not Specified
https://en.irct.ir/trial/55238	Ali Eshaghi	21/04/2021	Phase II	Recruiting	Iran	18-70	Healthy	Yes (17-35)
https://clinicaltrials.gov/ct2/show/NCT04813562	Fanyue Meng, Doctor	23/03/2021	Phase II	Recruiting	China	18-85	Healthy	Not specified
https://clinicaltrials.gov/ct2/show/NCT04765384	Janssen Vaccines & Prevention B.V.	12/03/2021	Phase II	Not yet recruiting	USA, Brazil, South Africa	18-45	Pregnant Women	Not specified
https://clinicaltrials.gov/ct2/show/NCT04822025	Szu-Min Hsieh, MD	01/05/2021	Phase II	Not yet recruiting	Taiwan	≥65	Healthy	Not specified
https://clinicaltrials.gov/ct2/show/NCT04824391	Zafer Sezer, Assoc.Prof.	10/02/2021	Phase II	Recruiting	Turkey	18-64	Healthy	Yes (18.5-32)
https://clinicaltrials.gov/ct2/show/NCT04748471	Odile LAUNAY, Professor	10/02/2021	Phase II	Not yet recruiting	France	≥18	Healthy	Not specified

https://clinicaltrials.gov/ct2/show/NCT04847050	Elad Sharon, M.D.	28/04/2021	Phase II	Recruiting	USA	≥18	Malignancy	Not specified
https://clinicaltrials.gov/ct2/show/NCT04728347	Jenny Low Guek Hong, MD	04/01/2021	Phase II	Recruiting	Singapore	≥18	Healthy	Not specified
https://ClinicalTrials.gov/show/NCT04655625	AnGes, Inc.	23/11/2020	Phase II/III	Active, not recruiting	Japan	≥18	Healthy	Not specified
CTRI/2020/08/027170	Dr Prasad Kulkarni	24/08/2020	Phase II/III	Active, not recruiting	India	18-99	Healthy	Not specified
https://ClinicalTrials.gov/show/NCT04672395	Vincent Mwangi, MD	17/12/2020	Phase II/III	Not yet recruiting	Belgium, Brazil, Colombia, Dominican Republic, Germany, Nepal, Panama, Philipines, Poland, South Africa	≥18	Healthy	Not specified
https://ClinicalTrials.gov/show/NCT04683224	Dr Gray Heppner	24/122020	Phase II/III	Not yet recruiting	Not specified	≥18	Healthy or stable pre-existing medical condition	Not specified
https://ClinicalTrials.gov/show/NCT04652102	CureVac AG	3/12/2020	Phase II/III	Recruiting	Belgium, Germany, Mexico, Netherlands, Peru, Spain	≥18	Healthy	Not specified
https://ClinicalTrials.gov/show/NCT04640233	Dr. Lalit Lakhwani, Contact: Dr. Agam Shah	23/111/2020	Phase II/III	Recruiting	India	≥18	Healthy	Not specified
https://ClinicalTrials.gov/show/NCT04642638	Mammen P. Mammen Jr, M.D.	24/11/2020	Phase II/III	Active, not recruiting	United States	≥18	Healthy	Not specified
https://ClinicalTrials.gov/show/NCT04400838	Andrew Pollard	28/05/2020	Phase II/III	Recruiting	United Kingdom	≥18	Relatively Healthy	Yes

https://clinicaltrials.gov/ct2/show/NCT04636697	Mary Hardison	19/11/2020	Phase II/III	Recruiting	USA, Canada, UK	≥18	Healthy and Co-morbidities	Yes BMI >30 Population 3 "high risk" co-morbidities. Pop 1 and 2 18.5-30
https://en.irct.ir/trial/54881	Mohammadreza Hosseinpour	14/03/2021	Phase II/III	Recruiting	Iran	18-75	Healthy	Not specified
https://clinicaltrials.gov/ct2/show/NCT04791423	Simone Lanini	15/03/2021	Phase II/III	Active, not recruiting	Italy	≥18	Healthy or mild comorbidities	Not specified
https://clinicaltrials.gov/ct2/show/NCT04754594	BioNTech SE/Pfizer	16/02/2021	Phase II/III	Recruiting	USA	≥18	pregnant	Not specified
https://clinicaltrials.gov/ct2/show/NCT04885764	Samia Girgis, Fatma Soliman E Ebeid	23/02/2021	Phase II/III	Recruiting	Egypt	≥18	Healthy	Not specified
https://clinicaltrials.gov/ct2/show/NCT04806529	Seqirus	15/12/2020	Phase II/III	Withdrawn	Australia	≥18	Healthy	Not specified
https://ClinicalTrials.gov/show/NCT04646590	Anhui Zhifei Longcom Biologic Pharmacy Co., Ltd.	16/12/2020	Phase III	Recruiting	China, Ecuador, Indonesia, Pakistan, Uzbekistan	≥18	Healthy	No
ISRCTN89951424	Dr Peter O'Reilly	05/01/2020	Phase III	Active, not recruiting	Brazil	18-55	Healthy	Not specified
https://ClinicalTrials.gov/show/NCT04516746	Ann Falsey, MD	11/08/2020	Phase III	Active, not recruiting	Argentina, Chile, Colombia, France, Peru, United States	18-130	Healthy	Not specified
https://ClinicalTrials.gov/show/NCT04540393	AstraZeneca	9/02/2020	Phase III	Suspended, SUSAR	Russian Federation	18-130	Healthy	Not specified
https://ClinicalTrials.gov/show/NCT04641481	Dr Krishna Mohan	23/11/2020	Phase III	Active, not recruiting	India	18-99	Healthy	Not specified
https://ClinicalTrials.gov/show/NCT04456595	Ricardo Palacios, MD, PhD	2/07/2020	Phase III	Active, not recruiting	Brazil	≥18	Healthy	Not specified

https://ClinicalTrials.gov/show/NCT04526990	Zhu Fengcai	26/08/2020	Phase III	Recruiting	Argentina, Chile, Mexico, Pakistan, Russian Federation	≥18	Healthy	Not specified
https://ClinicalTrials.gov/show/NCT04510207	Walid A Zaher, MD, MSc,PhD	12/08/2020	Phase III	Recruiting	Bahrain, Egypt, Jordan, United Arab Emirates	≥18	Healthy	Not specified
https://ClinicalTrials.gov/show/NCT04659239	Yasmin binti Mohamed Gani, PhD	9/12/2020	Phase III	Enrolling by invitation	Brazil, Malaysia	≥18	Healthy	Not specified
https://ClinicalTrials.gov/show/NCT04674189	Stephan Gehring	19/12/2020	Phase III	Recruiting	Germany	≥18	Healthy	Not specified
https://ClinicalTrials.gov/show/NCT04508075	Novilia Sjafri Bachtiar	11/08/2020	Phase III	Active, not recruiting	Indonesia	18-59	Healthy	Not specified
https://ClinicalTrials.gov/show/NCT04530396	Elena Smolyarchuk, Sergey Zyryanov	28/08/2020	Phase III	Active, not recruiting	Russia	18-111	Healthy	Not specified
https://ClinicalTrials.gov/show/NCT04642339	Alexis H García Piñero, MD	24/11/2020	Phase III	Not yet recruiting	Venezuela	≥18	Healthy	Not specified
https://ClinicalTrials.gov/show/NCT04656613	Mohamed Mostafa	7/12/2020	Phase III	Not yet recruiting	UAE	≥18	Healthy	Not specified
https://ClinicalTrials.gov/show/NCT04564716	Roman Plotnikov	25/09/2020	Phase III	Active not recruiting	Belarus	18 to 60	Healthy	Not specified
https://ClinicalTrials.gov/show/NCT04582344	Murat Akova, Prof; Serhat Ünal, Prof.	9/10/2020	Phase III	Recruiting	Turkey	18-59	Healthy	Not specified
https://ClinicalTrials.gov/show/NCT04614948	Janssen Vaccines & Prevention	4/11/2020	Phase III	Active, not recruiting	UK, Spain, South Africa, Phillipines, Germany, France, Colombia, Brazil, Belgium, US	≥18	Healthy	Not specified

https://ClinicalTrials.gov/show/NCT04505722	J Sadoff	10/08/2020	Phase III	Active, not recruiting	US, Argentina, Brazil, Chile, Colombia, Mexico, Peru, South Africa	≥18	Healthy	Not specified
https://ClinicalTrials.gov/show/NCT04560881	Pedro Cahn	16/09/2020	Phase III	Active, not recruiting	Argentina	18-85	Healthy	Not specified
https://ClinicalTrials.gov/show/NCT04470427	HM El Sahly, PB Gilbert, LR Baden	27/07/2020	Phase III	Active not recruiting	United States	≥18	Heightened risk individuals included	Yes
https://ClinicalTrials.gov/show/NCT04583995	Paul Heath	28/09/2020	Phase III	Recruiting	United Kingdom	18-84	Relatively Healthy	Yes
https://ClinicalTrials.gov/show/NCT04540419	Nikolay S. Dodonov	11/09/2020	Phase III	Active not recruiting	Russian Federation	18-85	Relatively Healthy	No (18.5-30)
https://ClinicalTrials.gov/show/NCT04651790	Katia Abarca	27/11/2020	Phase III	Recruiting	Chile	≥18	Relatively Healthy	Not specified
https://ClinicalTrials.gov/show/NCT04691908	Ilyas Kulmagambetov	25/12/2020	Phase III	Active not recruiting	Kazakhstan	≥18	Healthy	Not specified
ChiCTR2000034780	Nawal Al Kaabi	16/07/2021	Phase III	Active not recruiting	United Arab Emirates	≥18	Healthy	Not specified
ChiCTR2000039000	Abouqal Redouane	2/09/2020	Phase III	Recruiting	Morroco	≥18	Healthy	Not specified
https://ClinicalTrials.gov/show/NCT04617483	Hongxing Pan	31/10/2020	Phase III	Recruiting	China	≥18	Relatively Healthy	Not specified
https://ClinicalTrials.gov/show/NCT04612972	Coralith Garcia	9/09/2020	Phase III	Active not recruiting	Peru	≥18	Healthy	Not specified
ChiCTR2000039715	Fengcai Zhu	17/11/2020	Phase III	Recruiting	China	≥18	Healthy	Not specified
https://ClinicalTrials.gov/show/NCT04536051	Andrew Pollard	2/06/2020	Phase III	Recruiting	Brazil	≥18	Relatively Healthy	Not specified
https://clinicaltrials.gov/ct2/show/NCT04838795	Glenda E Gray, MBChB	18/02/2021	Phase III	Recruiting	South Africa	≥18 (healthcare workers)	Healthy	Not specified
https://clinicaltrials.gov/ct2/show/NCT04887207	Fengcai Zhu, Doctor	1/06/2021	Phase III	Not yet recruiting	Pending	≥18	Healthy	Not specified
https://clinicaltrials.gov/ct2/show/NCT04811664	Kathryn Stephenson	24/03/2021	Phase III	Recruiting	USA	18-26	Healthy	Not specified

https://clinicaltrials.gov/ct2/show/NCT04847102	Shuyuan Yang	28/05/2021	Phase III	Not yet recruiting	Pending	≥18	Healthy	Not specified
https://clinicaltrials.gov/ct2/show/NCT04852705	Guifan Li, M.S	1/05/2021	Phase III	Not yet recruiting	Pending	≥18	Healthy	Not specified
https://clinicaltrials.gov/ct2/show/NCT04741061	Elena Merkulova	19/02/2021	Phase III	Recruiting	Russia	≥18	Healthy	Not specified
https://clinicaltrials.gov/ct2/show/NCT04864561	Valneva Austria GmbH	26/04/2021	Phase III	Recruiting	UK	≥18	Healthy	Not specified
https://clinicaltrials.gov/ct2/show/NCT04780035	Rinat A. Maksyutov, PhD	18/11/2020	Phase III	Active, not recruiting	Russia	≥18	Healthy	Not specified
https://clinicaltrials.gov/ct2/show/NCT04860258	CureVac AG	22/04/2021	Phase III	Recruiting	Belgium	≥18 (comorbidities)	Comorbid	Not specified
https://clinicaltrials.gov/ct2/show/NCT04848467	Bayer Clinical Trials	10/05/2021	Phase III	Not yet recruiting	Argentina, Colombia, Peru	≥60	Healthy	Yes (18-35)
https://clinicaltrials.gov/ct2/show/NCT04805125	Heiner C. Bucher, Prof. Dr. med	19/04/2021	Phase III	Recruiting	Switzerland	≥18 (immunocompromised)	Immunocompromised	Not specified
https://clinicaltrials.gov/ct2/show/NCT04860297	Moderna Clinical Trials	16/04/2021	Phase III	Recruiting	USA	≥18 (transplant recipients)	Transplant	Not specified
https://clinicaltrials.gov/ct2/show/NCT04806113	Dr Ines Colmegna	11/03/2021	Phase III	Active, not recruiting	Canada	≥18	Immunosupressed	Not specified
https://clinicaltrials.gov/ct2/show/NCT04838847	CureVac AG	14/05/2021	Phase III	Not yet recruiting	Pending	≥18	Healthy	Not specified
https://clinicaltrials.gov/ct2/show/NCT04816669	Pfizer/BioNTech SE	1/04/2021	Phase III	Active, not recruiting	USA	18-55	Healthy	Not specified
https://rpcec.sld.cu/en/trials/RPCEC00000354-En	Maria Toledo Romani	8/03/2021	Phase III	Active, not recruting	Havana	19-80	Healthy	Not specified
https://rpcec.sld.cu/en/trials/RPCEC00000359-En	Maria del Carmen	22/03/2021	Phase III	Pending	Cuba, Guantanamo	19-80	Healthy	≥18 to ≤35
https://clinicaltrials.gov/ct2/show/NCT04747821	Ricardo Palacios, MD, PhD	7/02/2021	Phase IV	Active, not recruiting	Brazil	≥18	Healthy	Not specified
https://clinicaltrials.gov/ct2/show/NCT04789356	Marcus Lacerda, MD	18/03/2021	Phase IV	Recruiting	Brazil	18-49	Healthy	Not specified

https://clinicaltrials.gov/ct2/show/NCT04760132	Jens D Lundgren, MD	8/02/2021	Phase IV	Recruiting	Denmark	≥18	Healthy	Not specified
https://clinicaltrials.gov/ct2/show/NCT04754698	Kallas, MD, PhD	9/02/2021	Phase IV	Recruiting	Brazil	≥18 (autoimmune)	autoimmune	Not specified
https://clinicaltrials.gov/ct2/show/NCT04756830	Jose Cerbino	1/02/2021	Phase IV	Not yet recruiting	Brazil	≥18	Healthy	Not specified
https://clinicaltrials.gov/ct2/show/NCT04790851	Xiadong Sun	23/02/2021	Phase IV	Recruiting	China	≥18	Healthy	Not specified
https://clinicaltrials.gov/ct2/show/NCT04775069	Humanity & Health Medical Group Limited	15/03/2021	Phase IV	Not yet recruiting	Hong Kong	≥18 (chronic liver disease)	Liver Disease	Not specified
https://www.clinicaltrialsregister.eu/ctr-search/trial/2021-000930-32/BE	FPS Health-DGM	27/04/2021	Phase IV	Ongoing	Belgium	≥18 (haemodialysis)	Haemodialysis	Not specified
https://clinicaltrials.gov/ct2/show/NCT04780659	Soo Aleman, MD, PhD	23/02/2021	Phase IV	Recruiting	Sweden	≥18 (immunocompromised)	Immunocompromised	Not specified
https://clinicaltrials.gov/ct2/show/NCT04852861	Maria E Goossens MD PhD	10/05/2021	Phase IV	Enrolling by invitation	Belgium	18-55	Healthy	Not specified
https://clinicaltrials.gov/ct2/show/NCT04833101	Jing-Xin Li, PhD	7/04/2021	Phase IV	Active, not recruiting	China	≥18	Healthy	Not specified
